# Applying Permutation Tests and Multivariate Modification Indices to Configurally Invariant Models That Need Respecification

**DOI:** 10.3389/fpsyg.2017.01455

**Published:** 2017-08-24

**Authors:** Terrence D. Jorgensen

**Affiliations:** Research Institute for Child Development and Education, University of Amsterdam Amsterdam, Netherlands

**Keywords:** configural invariance, permutation tests, measurement equivalence/invariance, confirmatory factor analysis, Lagrange multipliers, modification indices

## Abstract

The assumption of equivalence between measurement-model configurations across groups is typically investigated by evaluating overall fit of the same model simultaneously to multiple samples. However, the null hypothesis (H_0_) of configural invariance is distinct from the H_0_ of overall model fit. Permutation tests of configural invariance yield nominal Type I error rates even when a model does not fit perfectly (Jorgensen et al., 2017, in press). When the configural model requires modification, lack of evidence against configural invariance implies that researchers should reconsider their model's structure simultaneously across all groups. Application of multivariate modification indices is therefore proposed to help decide which parameter(s) to free simultaneously in all groups, and I present Monte Carlo simulation results comparing their Type I error control to traditional 1-*df* modification indices. I use the Holzinger and Swineford (1939) data set to illustrate these methods.

Many behavioral researchers do not have the luxury of being able to directly observe the phenomena they study. For example, organizational researchers need to measure job satisfaction or morale. Clinicians need to measure various psychological disorders. Social psychologists and sociologists need to measure attitudes and social orientations. Educational researchers need to measure teaching and learning outcomes. Often, researchers rely on indirect measures, such as self-report scales, and psychometric tools, such as reliability estimates and latent trait models [e.g., confirmatory factor analysis (CFA) and item-response theory (IRT) models] facilitate evaluation of the quality of those measurements.

Similarly frequent is the need for researchers to compare groups, in either experimental (e.g., treated vs. control) or observational contexts (e.g., demographic or intact groups). In order to make valid comparisons of scale responses across groups, the scale must function equivalently for those groups. In other words, if measurement parameters are equivalent across groups, observed group means will only differ as a function of differences on the latent trait itself (Meredith, 1993). Measurement equivalence/invariance (ME/I) has received a great deal of attention in the methodological literature, so I provide only a cursory introduction here; interested readers are encouraged to find more in-depth discussion in Meredith (1993); Reise et al. (1993); Vandenberg and Lance (2000), and Putnick and Bornstein (2016).

Latent trait models facilitate the investigation of ME/I, and different levels of ME/I have been defined according to categories of model parameters. In a CFA framework, configural invariance is represented in a model with the same pattern of fixed and free (i.e., near-zero and substantial) factor loadings across groups, although the values of these parameters may differ across groups. When fitting models to multivariate normally distributed data using maximum likelihood estimation, the null hypothesis (H_0_) of configural invariance is traditionally tested using a likelihood-ratio test statistic (LRT)^1^, which is distributed as a χ^2^ random variable with *df* equal to the number of nonredundant observed means and (co)variances minus the number of estimated model parameters. Configural invariance is the least restrictive level of ME/I, so it can be used as a baseline model for comparing more restrictive assumptions of ME/I, which are represented by models that are nested within the configural model.

Metric equivalence (or “weak” invariance) indicates the additional assumption that the values of factor loadings are equal across groups, and this assumption must hold in order to make valid across-group comparisons of latent variances or correlations. This model is nested within the configural model, so a Δχ^2^ test can be used to test the H_0_ of exact metric equivalence. If a researcher concludes that full (or partial^2^) metric equivalence holds, that model is used as a baseline model to test scalar equivalence (or “strong” invariance”) by additionally constraining indicator intercepts (or thresholds for binary or ordinal indicators) to equality across groups. Scalar invariance is required for valid comparisons of latent means to be made. Researchers can also test homogeneity of residual variances across groups (“strict” invariance), but because that assumption is not required for valid comparisons of latent parameters, it is not tested as often (Putnick and Bornstein, 2016).

The current paper discusses recent advances only in tests of configural invariance, which is the least restrictive level of invariance. A false H_0_ would imply that model configurations differ across groups, in which case data-generating population processes do not share all the same parameters across groups. A test that rejects the H_0_ of configural invariance would therefore prohibit researchers from testing more restrictive levels of ME/I. Currently, configural invariance is assessed by evaluating the overall fit of the configural model (Putnick and Bornstein, 2016). A significant LRT or fit indices that do not meet criteria for adequate fit (Hu and Bentler, 1999) would be expected when configural invariance does not hold across populations, because the hypothesized model could only represent the data-generating process for one (subset of) group(s), and would be incorrect for at least one of the other groups; thus, the poor fit of the model to that group would be reflected in the overall model fit measures. However, the fact that a false H_0_ should lead to a poor fit does not imply the reverse^3^: If the model fits poorly, that does not necessarily imply that true population models are configurally noninvariant. A hypothesized configural model could fit poorly for a different reason; specifically, the true data-generating process might be equivalent across groups (i.e., H_0_ of configural invariance is true), but the specified model is a poor approximation of the true functional form of that process (i.e., false H_0_ that the model is correctly specified).

Using a newly proposed permutation test of configural invariance (Jorgensen et al., 2017, in press), the H_0_ of configural invariance can be tested with nominal Type I error rates even when the H_0_ of correct specification is false. I extend this line of research by proposing the use of multivariate modification indices (Bentler and Chou, 1992) to guide researchers in respecifying their inadequately fitting configural models when there is no evidence against the H_0_ of group equivalence in true model configurations. This study is therefore only concerned with the situation when the H_0_ of configural invariance is true (but the model does not fit well), not when the H_0_ is false. To evaluate the use of multivariate modification indices for the purpose of testing whether the same parameter should be freed simultaneously across groups, I designed a small-scale simulation study as a proof of concept to show that they are capable of preventing Type I error inflation better than traditional 1-*df* modification indices, which test parameters in only one group at a time rather than simultaneously across all groups.

I begin by reviewing in more detail issues with testing model fit vs. configural invariance, using an analysis of the classic Holzinger and Swineford (1939) dataset to demonstrate the use of the permutation test and to illustrate the implication of a configurally invariant model that requires respecification. I then introduce Bentler and Chou's (1992) multivariate extension of modification indices, which are recently available in the open-source lavaan package (Rosseel, 2012) for structural equation modeling (SEM) in R (R Core Team, 2017), and discuss how they can be used in the context of respecifying a multigroup model in a way consistent with the H_0_ of configural invariance. I then describe the small-scale Monte Carlo simulation study comparing Type I error rates using univariate and multivariate modification indices. I conclude with recommendations for future applied and methodological research.

## Issues with model-fit tests of configural invariance

Configural invariance in a multigroup context is equivalence in model configurations across the populations of interest. The analysis models are typically specified as configurally invariant, and the LRT of overall model fit is used to evaluate whether the model adequately approximates the population models. As noted in the Introduction, rejection of the H_0_ of exact model fit could imply numerous conditions, including but not limited to the following: (a) the hypothesized model corresponds well to one or more populations but poorly to at least one other; (b) the model does not correspond to any group's model, for different reasons across groups; (c) all groups true models are configurally invariant, but the hypothesized model does not correspond to that shared functional form. Thus, when a model's overall fit to multiple groups needs improvement, the decision of how to respecify the model would depend on which condition led to poor overall fit.

Because the LRT is a test of overall exact fit of the model to the data, two potential sources of misspecification are confounded (Cudeck and Henly, 1991; MacCallum, 2003): estimation discrepancy (due to sampling error) and approximation discrepancy (due to a lack of correspondence between the population and analysis models). Because configural invariance is assessed by testing the absolute fit of the configural model, the LRT for a multigroup model further confounds two sources of approximation discrepancy (Jorgensen et al., 2017, in press): the overall discrepancy between population and analysis models could be partitioned into (a) differences between groups' true population models and (b) discrepancies between each group's population and analysis models. The H_0_ of configural invariance only concerns the former source of approximation discrepancy (which I will refer to as *group discrepancy*), whereas the latter source is an issue of model-fit in general (which I will refer to as *overall approximation discrepancy*).

Good model fit and equivalent model configurations are both important foundational assumptions of ME/I because testing equality of measurement parameters is only valid if the estimated parameters correspond to actual parameters of the true data-generating process. But merely testing the overall fit of a configural model does not provide adequate information about whether model configurations can be assumed equivalent across groups. It is possible (perhaps even probable) that a model provides as good a description of one population as it does for another population (e.g., men and women or respondents from different countries), even if the model fits poorly or only approximately well. Evaluating overall fit therefore tests the wrong H_0_ by confounding group equivalence and overall exact model fit into a single test. The permutation method introduced by Jorgensen et al. (2017, in press) disentangles group discrepancy from overall approximation discrepancy.

Another common issue with model-fit evaluation is the common perception that the LRT nearly always rejects good models because SEM requires large sample sizes for estimation. Although it is true that power is a function of sample size, an analysis model that corresponds perfectly with a true population model would not yield inflated Type I errors (actually, small-sample bias would; Nevitt and Hancock, 2004) because the H_0_ would be true. But because theoretical models are more realistically interpreted as approximations to more complex population models (MacCallum, 2003), the H_0_ of exact fit should rarely be expected to be precisely true in practice. In order to help researchers evaluate the degree to which a H_0_ is false, numerous indices of approximate fit have been proposed since the 1970s, analogous to providing standardized measures of effect size that accompany a null-hypothesis significance test in other contexts (e.g., Cohen's *d* to accompany a *t*-test result).

Unfortunately, approximate fit indices (AFIs) or their differences (Δ) between competing models rarely have known sampling distributions. Even when they do [e.g., the root mean-squared error of approximation (RMSEA); Steiger and Lind, 1980], it is often unclear how to interpret the magnitude of a (Δ)AFI. Researchers frequently rely on rule-of-thumb cutoffs, such as those proposed by Hu and Bentler (1999) for AFIs or by Cheung and Rensvold (2002) for ΔAFIs, either based on intuition or derived from simulation studies under specific conditions that might not generalize to the wide array of SEMs encountered in practice. Although it is reasonable to argue that models with only negligible misspecifications should not be rejected, it is unreasonable to expect a single rule-of-thumb cutoff for any (Δ)AFI to perform consistently across various models (Cheung and Lau, 2012; Pornprasertmanit et al., 2013).

Putnick and Bornstein (2016) found that 45.9% of studies they reviewed supplemented the LRT with at least one (Δ)AFI to draw conclusions about various levels of ME/I. Given the popularity of (Δ)AFIs, it is safe to assume any of those researchers who reported a significant LRT still did not reject their model if the (Δ)AFI(s) were within the guidelines of acceptable fit. The LRT appears to be used as the sole criterion to evaluate ME/I only half as often (16.7%) as (Δ)AFI(s) alone (34.1%), the most popular of which is the comparative fit index (CFI; Bentler, 1990), at least in the context of ME/I (Putnick and Bornstein, 2016). Given the sensitivity of (Δ)AFI sampling distributions to data and model characteristics (Marsh et al., 2004), basing conclusions about configural invariance on AFIs (e.g., interpreting CFI >0.95 as evidence of good approximate fit) leads to Type II errors in large samples, but can also lead to inflated Type I errors in small samples (Jorgensen et al., 2017). Permutation also provides a solution to problems with unknown (Δ)AFI sampling distributions by comparing observed configural-model AFIs to empirical sampling distributions derived under the H_0_ of equivalent group configurations (Jorgensen et al., in press).

## Illustrative example

To demonstrate the utility of the recently proposed permutation test and how multivariate modification indices can be used to modify a model under the assumption of configural invariance, I fit a three-factor multigroup CFA model with simple structure to the Holzinger and Swineford (1939) dataset, which has often been repurposed for illustrative examples (e.g., Jöreskog, 1969; Tucker and Lewis, 1973). A subset of the data are available as part of the lavaan package (Rosseel, 2012), including three indicators for each of three mental-ability constructs: visual, textual, and speed. This illustration assesses configural invariance across two schools (Pasteur: *N* = 156; Grant–White: *N* = 145), which is the most common number of groups analyzed (75%; Putnick and Bornstein, 2016). I provide R syntax for all analyses in the Appendix, and Table 1 presents descriptions of indicators of each factor, as well as parameter estimates from the configural CFA model.

**Table 1:** Estimated parameters from CFA with simple structure.

**Common factor**	**Indicator**	**Mental-Ability test description**	**Pasteur School**		**Grant–White School**	
			**λ**	**θ**	**λ**	**θ**
Visual	*X*_1_	Visual perception	1.047	0.298	0.777	0.715
	*X*_2_	Cubes	0.412	1.334	0.572	0.899
	*X*_3_	Lozenges	0.597	0.989	0.719	0.557
Textual	*X*_4_	Paragraph comprehension	0.946	0.425	0.971	0.315
	*X*_5_	Sentence completion	1.119	0.456	0.961	0.419
	*X*_6_	Word meaning	0.827	0.290	0.935	0.406
Speed	*X*_7_	Speeded addition	0.591	0.820	0.679	0.600
	*X*_8_	Speeded counting of dots	0.665	0.510	0.833	0.401
	*X*_9_	Speeded discrimination between straight and curved capital (uppercase) letters	0.545	0.680	0.719	0.535

*λ, factor loading; θ, residual variance. Factor variances were fixed to 1. Saturated mean structure not presented. In the Pasteur school, visual–textual covariance = 0.484, visual–speed covariance = 0.299, and speed–textual covariance = 0.325. In the Grant–White school, visual–textual covariance = 0.541, visual–speed covariance = 0.523, and speed–textual covariance = 0.336. SEs not reported, but all parameters significantly differed from zero at α = 5%*.

There is evidence that the configural model does not fit the data perfectly, $\chi_{\left(48\right)}^{2}$ = 115.85, *p* = 0.0000002, and both CFI = 0.923 and RMSEA = 0.097, 90% CI [0.075, 0.120], suggest that the degree of misspecification is not ignorable, using Hu and Bentler's (1999) recommended cutoffs of CFA >0.95 and RMSEA <0.06. Thus, the three-factor model with simple structure does not appear to adequately capture features of the data-generating process. Without additional information about group discrepancy, a researcher interested in modifying the model might begin by assessing model fit separately within each group. Similar results would be found for both the Pasteur school, $\chi_{\left(24\right)}^{2}$ = 64.31, *p* = 0.00002, CFI = 0.903, RMSEA = 0.104, 90% CI [0.074, 0.135], and the Grant–White school, $\chi_{\left(24\right)}^{2}$ = 51.54, *p* = 0.001, CFI = 0.941, RMSEA = 0.089, 90% CI [0.055, 0.122], leading to the conclusion that both groups' models require modification. But without informing the researcher about (lack of) evidence of group discrepancy, it would be unclear whether the most appropriate course of action would be to attempt freeing the same parameter(s) in both groups simultaneously or to modify each group's model independently.

### Permutation test

A permutation test of configural invariance can be conducted by comparing $\chi_{\left(48\right)}^{2}$ = 115.85 to an empirical sampling distribution rather than a central χ^2^ distribution with 48 *df*. An empirical sampling distribution under the H_0_ of equivalent model configurations can be estimated by randomly reassigning rows of data to the two schools, fitting the configural model to the permuted data, and saving χ^2^. Repeating these steps numerous times results in a permutation distribution of χ^2^, and a *p* value can be calculated as the proportion of the distribution that exceeds (indicates worse fit than) the observed χ^2^. Because the students are assumed equivalent when they are randomly reassigned to schools, the permutation distribution reflects the sampling variance of χ^2^ under the assumption that the schools share the same data-generating model, but without assuming that the data-generating model corresponds perfectly with the fitted model. Due to poor model fit (i.e., the H_0_ of no overall approximation discrepancy is rejected), the permutation distribution is not expected to approximate a central χ^2^ distribution with 48 *df*, but it has been shown to approximate the sampling distribution under the H_0_ of no group discrepancy (Jorgensen et al., 2017, in press). Likewise, CFI and RMSEA can be compared to permutation distributions, overcoming important limitations of AFIs: the lack of a theoretical sampling distribution for CFI, and the lack of consensus about a particular value of CFI or RMSEA that would indicate adequate approximate fit in all contexts.

A permutation test revealed no evidence against the H_0_ of configural invariance using either χ^2^(*p* = 0.19), CFI (*p* = 0.17), or RMSEA (*p* = 0.19) as criterion. Thus, model modification can proceed by freeing the same parameter(s) in both groups simultaneously. This could minimize well documented problems with data-driven use of modification indices leading to models that do not generalize to new samples from the same population (MacCallum, 1986; MacCallum et al., 1992; French and Finch, 2008). The hypothesized CFA model fixes 18 cross-loadings and 36 residual covariances to zero in each of two groups, resulting in 108 modification indices for individual parameters (i.e., 1-*df* tests). Inspecting multivariate modification indices (i.e., 2-*df* tests) reduces the number of tests by half, from 108 to 54. More generally, with *g* groups, there will always be *g* times as many 1-*df* modification indices as *g*-*df* modification indices. Before presenting results for the CFA model, I elaborate further on the multivariate modification index.

### Multivariate modification indices

My discussion below is in the context of maximum likelihood estimation, but the same concepts can be applied to other discrepancy functions for estimating SEM parameters (Bentler and Chou, 1992). Lagrange multipliers fit into a framework of three tests of parameter restrictions, including Wald tests and nested-model LRTs (Buse, 1982). The LRT requires fitting both a restricted (M_0_) and unrestricted (M_1_) model. The LRT statistic is calculated by comparing the log-likelihood (ℓ) of the data under each model: LRT = −2 × (ℓ _0_ − ℓ _1_). If the H_0_ is true and distributional assumptions are met, the LRT statistic is asymptotically distributed as a central χ^2^ random variable with *df* equal to the number of restrictions in M_0_ relative to M_1_.

The Wald and Lagrange multiplier tests are asymptotically equivalent to the LRT, but the Wald test only requires fitting M_1_, whereas the Lagrange multiplier test only requires fitting M_0_ (for details see Buse, 1982). The modification indices provided by most SEM software packages are 1-*df* Lagrange multipliers associated with each fixed parameter (or equality constraint), and they estimate the LRT statistic (i.e., the change in χ^2^ of M_0_) if that constraint were freed in M_1_ (but without needing to fit M_1_), assuming all other parameter estimates would remain unchanged between M_0_ and M_1_. Calculation of Lagrange multipliers utilizes information from the gradient (first derivative of the discrepancy function). Specifically, the curvature of the likelihood function evaluated with respect to the null-hypothesized value (θ_0_) of a fixed parameter (typically zero) provides a clue about how far θ_0_ is from the true θ, relative to the estimated sampling variability.

Bentler and Chou (1992) extended this simple idea to evaluating the curvature of the likelihood function in multiple dimensions with respect to a vector of constrained parameters. Multivariate Lagrange multipliers have only been implemented in some SEM software packages, such as EQS (Bentler, 2006) and PROC CALIS (SAS Institute Inc., 2011). In the spirit of the open-access *Frontiers* journal^4^, my applied example utilizes the freely available open-source R package lavaan (Rosseel, 2012), which implements multivariate Lagrange multipliers via the lavTestScore() function, along with the widely available 1-*df* statistics via the modificationIndices() function. I discuss both in the context of the example CFA applied to the Holzinger and Swineford (1939) data set. As noted in previous research (e.g., MacCallum et al., 1992) and SEM textbooks (e.g., Brown, 2015; Kline, 2015), purely data-driven specification searches do not lead to generalizable, reproducible models, so model modifications should always be guided by substantive theory. The current study, however, is focused on the statistics themselves, so my interpretation of results focuses primarily on decisions that a hypothetical researcher might be influenced to make when inspecting modification indices.

Table 2 presents the largest 1-*df* modification indices from the CFA model with simple structure, six of which (three in each group) were above 10. These results do not provide unambiguous guidance about which parameter constraints should be released. The largest modification index is associated with a residual covariance between the seventh and eighth indicators (of the same factor) in the Grant–White group. The second largest modification index (very similar in value to the largest) is associated with a cross-loading of the ninth indicator (speeded discrimination between straight and curved letters) on the visual factor, also in the Grant–White group. This is also the only parameter that is significant for both groups, although it is not significant in the Pasteur group after a Bonferroni adjustment for multiple tests. Arguably, it may make theoretical sense to free this parameter given that the *X*_9_ task required similar visual skills as the other visual indicators. If one considered the standardized expected parameter changes in tandem with modification indices, as advised by Saris et al. (2009) see also Whittaker (2012), then the cross-loading of the first indicator on the textual factor in the Pasteur group might be considered the best candidate instead.

**Table 2:** Largest univariate and multivariate modification indices for fixed (to zero) parameters.

**School**	**Parameter**	**MI**	**EPC**	**SEPC**
Pasteur	Visual → *X*_9_	11.07^*a*^	0.32	0.32
	Textual → *X*_1_	10.18^a^	0.89	0.76
	*X*_4_ ↔ *X*_6_	11.28^a^	−0.33	−0.29
Grant–White	Visual → *X*_7_	11.27^a^	−0.39	−0.38
	Visual → *X*_9_	24.54^a^^,^^b^	0.58	0.57
	*X*_7_ ↔ *X*_8_	24.82^a^^,^^b^	0.61	0.57
Multivariate	Visual → *X*_7_	16.45^a^^,^^b^		
(MI = ${\hat{\chi }}_{df=2}^{2}$)	Visual → *X*_9_	35.61^a^^,^^b^		
	*X*_7_ ↔ *X*_8_	29.01^a^^,^^b^		

*MI, modification index. (S); EPC, (standardized) expected parameter change (unavailable for multivariate MIs). → indicates a factor loading. ↔ indicates a covariance*.

a *Significant at α = 5%*.

b *Significant at Bonferroni-adjusted α = 0.05/108 = 0.00046 (critical ${\hat{\chi }}_{df=1}^{2}$ = 12.26) for 1-df MIs, or α = 0.05/54 = 0.00093 (critical ${\hat{\chi }}_{df=2}^{2}$ = 10.97) for 2-df MIs*.

The bottom rows of Table 2 also present the significant 2-*df* modification indices, the largest of which was for the cross-loading of the ninth indicator on the visual factor, which was also the only parameter with a large 1-*df* modification index in both groups. The interpretation of these tests is less ambiguous because they formally test the same parameter constraint simultaneously in both groups, which the permutation test implied is appropriate because there is no evidence the group configurations differ. Freeing this parameter did lead to significantly better model fit, $\Delta \chi_{\left(2\right)}^{2}$ = 34.31 (comparable to the expected χ^2^ = 35.61 in Table 2), *p* = 0.00000004, although the modified model still did not fit perfectly, $\chi_{\left(46\right)}^{2}$ = 81.55, *p* = 0.001, CFI = 0.960, RMSEA = 0.072, 90% CI [0.045, 0.097]. Because the purpose of this application is merely to demonstrate tools for testing and modifying configural models, I do not consider further modifications of the example CFA.

Next, I present a small-scale simulation study designed to evaluate the use of multivariate modification indices. A concise simulation was designed to keep the focus on the purpose of this simulation, which is to provide a “proof of concept” that multivariate modification indices can control Type I errors better than univariate modification indices when the hypothesized model is approximately well specified but needs improvement. I focus on this situation because modification indices are unlikely to lead to the true data-generating model when a hypothesized model deviates substantially from it (MacCallum, 1986; MacCallum et al., 1992), and there is no reason to expect multivariate modification indices to perform differently in the latter situation.

## Methods

To simulate data in which the H_0_ of configural invariance was true but the H_0_ of exact model fit is false, I specified a two-factor CFA model for four groups, with three indicators for each of two common factors. The factor loadings were λ = 0.6, 0.7, and 0.8 for the first, second, and third indicator of each factor, respectively. The residual variances were specified as 1 – λ^2^ so that indicators were multivariate normal with unit variances. Factor variances were fixed at 1 (also in the analysis model, for identification), and all indicator and factor intercepts were zero. Factor correlations were 0.2, 0.3, 0.4, and 0.5 in Groups 1, 2, 3, and 4, respectively, so that population covariance matrices were not identical, although model configurations were equivalent.

Imperfect overall model fit was specified by setting two residual covariances in the four populations with values of 0.2 between the first and fourth indicators, corresponding to a moderate residual correlation of 0.2/0.64 = 0.31, and 0.15 between the second and fifth indicators, corresponding to a moderate residual correlation of 0.15/0.51 = 0.29. These parameters were specified in all groups, so the population models were configurally invariant. Fixing these two residual covariances to zero in the analysis model resulted in significant misfit, $\chi_{\left(32\right)}^{2}$ = 54.05, *p* = 0.009, when the model was fit to the population covariance matrices, using samples sizes of *N* = 100 in each group. Approximate fit was questionable, acceptable CFI = 0.962, unacceptable RMSEA = 0.083, 90% CI [0.042, 0.120], so the configural model would have a considerable chance of being rejected when fit to a random sample drawn from this population. These fit measures are from the results of fitting the model to the population rather than sampled data, so they give an indication of the fit of the model, free from sampling error.

The configural model fixed six cross-loadings and 15 residual covariances to zero, yielding 21 modification indices to consider in each of four groups. The Bonferroni-adjusted α level was therefore 0.05/21 = 0.0024 for 4-*df* simultaneous tests and 0.05/84 = 0.0006 for 1-*df* tests; unadjusted α levels were not considered. I generated 1,000 random samples of *N* = 100 from each of the populations specified above, fit the configural model to the data, and recorded decisions about overall model fit (χ^2^, CFI, and RMSEA) and model respecification (univariate and multivariate modification indices). Within each replication, I also used a permutation test of configural invariance. When the model needed respecification, the parameter with the largest significant 4-*df* modification index was freed in all groups, iteratively until no modification indices were significant. A replication was flagged for having made a familywise Type I error if in any iteration, the largest significant 4-*df* modification index belonged to any parameter besides the two omitted residual covariances; correct detections of the omitted parameters were also flagged to calculate power. Parameters were not freed on the basis of univariate modification indices, but I also recorded whether the largest significant 1-*df* modification index in the first iteration belonged to any parameter besides the two omitted residual covariances, as a basis for comparing the familywise Type I error rates of 4-*df* modification indices to a lower-bound for the familywise Type I error rates of 1-*df* modification indices.

## Results

Using overall model fit as the criterion for evaluating configural invariance led to rejecting the model in 99.9% of replications using a significant LRT as criterion. Using Hu and Bentler (1999) criterion for approximate model fit, the model was rejected in 93.9% of replications by CFI < 0.95 and 100% using RMSEA > 0.06. Thus, researchers using any of these criteria would frequently be motivated to modify their configural model. Knowing whether the data showed evidence of equivalent model configurations (despite poor fit) would therefore be very useful. The permutation test falsely rejected the H_0_ of configural invariance in only 4.9% of the 1,000 replications, so the Type I error rate did not deviate substantially from the nominal α = 5%. This demonstration is consistent with previous results investigating the permutation method for testing ME/I in a two-group scenario (Jorgensen et al., 2017, in press). The unique contribution of this simulation, however, is to evaluate the performance of rarely utilized multivariate modification indices.

Multivariate modification indices correctly detected that at least one of the two omitted residual covariances should be freed in 99.6% of the replications, and correctly detected both omitted parameters in 73.9% of replications. This was accomplished while maintaining nominal (4.4%) familywise Type I errors across iterative modifications. By comparison, the largest 1-*df* modification index in the original configural model flagged an incorrect parameter in 9.5% of replications, implying that familywise Type I error rates would be at least that bad if they were instead used to iteratively modify the model. The poor performance of decisions based solely on 1-*df* modification indices is also consistent with previous results (MacCallum, 1986; MacCallum et al., 1992).

## Discussion

The aim of this paper was to advance two methods for testing configural invariance: how to test the correct H_0_ and how to test constraints in a poor-fitting configural model. A recently developed tool is a permutation test of the H_0_ of equivalent model configurations, which has shown promising control of Type I errors even when a configural model fits poorly (Jorgensen et al., 2017, in press). When the data show no strong evidence against the H_0_, researchers might be motivated to explore ways to modify their model to better reflect the data-generating process. Multivariate Lagrange multipliers (Bentler and Chou, 1992) can provide tests of constraints on the same parameter simultaneously across groups. A small-scale simulation illustrated how these could limit Type I errors better than traditional 1-*df* modification indices for individual fixed parameters within each group.

The simulation was not designed to provide comprehensive information across a variety of conditions, but it contributes some evidence that these tools warrant further investigation. Given that fully invariant metric (17.8%) and scalar (42.2%) models are rejected many times more often than configural (5.5%) models (Putnick and Bornstein, 2016), it is easier to find guidance in the literature about modifying metric and scalar models to establish partial invariance (e.g., Byrne et al., 1989; Vandenberg and Lance, 2000; Millsap, 2011). The current study therefore contributes to a sparser literature on modifying configural models, which Jorgensen et al. (2017, in press) showed might require more careful attention than common practice currently pays it. Note, however, that the current investigation does not address the issue of establishing “partial configural” invariance, but rather improving the fit of a configurally invariant model. More extensive investigations could shed light on the general applicability of the permutation test and of multivariate modification indices across a variety of conditions (e.g., different numbers of groups, sample sizes and ratios, varying other nonzero parameter values). For instance, the Holzinger and Swineford (1939) example application had only two groups, which may not be as prone to inflated Type I error rates as the four-group simulated data showed for 1-*df* modification indices.

This paper focused only on the situation when the H_0_ of configural invariance was true. When the data provide evidence against the assumption of equivalent model configurations^5^, more restrictive levels of invariance cannot be assumed either, nor would the proposed use of multivariate modification indices be relevant for modifying the model simultaneously across groups. If there are more than two groups, one could potentially test whether each pair of groups provide evidence against configural invariance, then test more restrictive levels of ME/I only for subsets that do not. Future research would be required to reveal whether Type I error rates could be maintained under such a follow-up procedure, but Jorgensen et al. (2017, in press) did find nominal error rates for the omnibus test of configural invariance with two-group data. According to Putnick and Bornstein (2016), most studies (75%) involve only two groups, so follow-up tests on subsets of groups might not be required often in practice.

I conclude by reiterating the importance of substantive theory to guide the process of model respecification (Brown, 2015; Kline, 2015). Purely data-driven use of modification indices tends to result in models that are over-fit to sample-specific nuances rather than mimicking the true data-generating process (MacCallum, 1986; MacCallum et al., 1992). Modification indices only tend to identify the correct parameter(s) to free when the model is already close to correctly specified, not when the model deviates substantially in form from the true model (MacCallum, 1986; MacCallum et al., 1992), so the same behavior should be expected from the multivariate modification indices applied to simultaneous changes in a single model across groups. Assuming the configural model is close to correctly specified, expected parameter changes may also provide useful supplementary information to use in tandem with modification indices (Saris et al., 2009; Whittaker, 2012), but like modification indices, their validity rests on the assumption that the structure of the model is basically correct except that at least one parameter constraint is not near its true population value. Hayduk (2014) showed that this may not be a safe assumption, given that factor models can fit data patterns from very different kinds of models, so poorly fitting factor models might be misspecified in ways beyond fixing too many parameters to zero. Correlation residuals provide information about model inadequacy in terms of the data pattern that the model tries to reproduce, so their inspection might be more likely to help a researcher speculate about different kinds of data-generating models. However, Lagrange multipliers are useful for testing specific hypotheses about parameter constraints, which are asymptotically equivalent to a LRT but only require fitting the constrained model rather than many less restricted models.

## Author contributions

TJ is responsible for the data analysis (using openly available data), design the simulation study, and writing the manuscript.

### Conflict of interest statement

The author declares that the research was conducted in the absence of any commercial or financial relationships that could be construed as a potential conflict of interest.

## Footnotes

^1^Although the configural model is only tested with a single model's χ^2^ statistic, this statistic is nonetheless equal to −2 times the difference between log-likelihoods of models representing two competing hypotheses: the hypothesized configural model (labeled H0 in the output from software such as M*plus* and lavaan) and the saturated model (labeled H1, representing the default alternative hypothesis of a completely unrestricted model). Because the saturated model has χ^2^ and *df* = 0, a Δχ^2^ test between the configural and saturated models would therefore be calculated by subtracting zero from the configural model's χ^2^ and *df*, yielding the same values.

^2^Partial invariance models posit that some, but not all, measurement parameters can be constrained to equality across groups or occasions, which still allows valid comparisons of latent parameters across groups Byrne et al., 1989.

^3^This logical fallacy is referred to as *affirming the consequent*, and has the general form: A implies B, B is true, therefore A is true. This is demonstrably invalid using simple examples for which it is false, such as: “If today is Saturday, it is the weekend. It is in fact the weekend; therefore it is Saturday.” The fact that it is the weekend does not imply it is Saturday because it could also be Sunday; there are multiple conditions that could lead to the same state.

^4^As stated on the Frontiers web page (http://home.frontiersin.org/about/about-frontiers): “Our grand vision is to build an Open Science platform where everybody has equal opportunity to seek, share and generate knowledge, and that empowers researchers in their daily work.”

^5^See Jorgensen et al. (2017; in press) for an investigation of power to detect different model configurations.

## Appendix

**R Syntax for Applied Example**

## use data available in lavaan package
library(lavaan)
HS <- lavaan::HolzingerSwineford1939
## specify configural invariance model
mod.config <- '
visual =~ x1 + x2 + x3
textual =~ x4 + x5 + x6
speed =~ x7 + x8 + x9
'
## fit model to schools, print results
fit.config <- cfa(mod.config, data = HS, std.lv = TRUE, group = “school”)
summary(fit.config, fit = TRUE)
fitMeasures(fit.config, c(“chisq”,“df”,“pvalue”,“cfi”,“rmsea”,“rmsea.ci.lower”,
“rmsea.ci.upper”))
## fit model separately per school
fit.Pasteur <- cfa(mod.config, data = HS[HS$school = = “Pasteur”,], std.lv = TRUE)
fitMeasures(fit.Pasteur, c(“chisq”,“df”,“pvalue”,“cfi”,“rmsea”,
“rmsea.ci.lower”,“rmsea.ci.upper”))
fit.Grant <- cfa(mod.config, data = HS[HS$school = = “Grant-White”,], std.lv = TRUE)
fitMeasures(fit.Grant, c(“chisq”,“df”,“pvalue”,“cfi”,“rmsea”,
“rmsea.ci.lower”,“rmsea.ci.upper”))
## Permutation Test using lavaanList()
set.seed(3141593)
dataList <- lapply(1:200, function(i) {HS$school<- sample(HS$school); HS})
out.site <- cfaList(mod.config, dataList = dataList, std.lv = TRUE, store.slots = NULL, group = “school”, FUN = function(x) lavaan::fitMeasures(x, c(“chisq”,“cfi”,“rmsea”)), parallel = “snow”, ncpus = 3, iseed = 3141593)
PF <- as.data.frame(do.call(rbind, out.site@funList))
OF <- fitMeasures(fit.config, c(“chisq”,“cfi”,“rmsea”))
mean(PF[[“chisq”]] > OF[“chisq”])
mean(PF[[“cfi”]] <OF[“cfi”])
mean(PF[[“rmsea”]] > OF[“rmsea”])
## Permutation Test also available in the semTools package
# library(semTools)
# permuteMeasEq(nPermute = 200, con = fit.config, AFIs = c(“chisq”,“cfi”,“rmsea”))
## inspect univariate (1-df) and multivariate (2-df) modification indices
MI1 <- modindices(fit.config)
MI1$p.value <- pchisq(MI1$mi, df = 1, lower.tail = FALSE)
MI1$bonf <- p.adjust(MI1$p.value, method = “bonferroni”)
MI1[MI1$mi > 10,]
MI1[MI1$bonf <0.05,]
## multivariate tests require changing the lavTestScore() source code in lavaan.
## Source code for the myScoreTest() function is available from the author on request.
MI2 <- do.call(rbind, lapply(unique(paste0(MI1$lhs, MI1$op, MI1$rhs)), function(x) {out<- myScoreTest(fit.config, add = x, univariate = FALSE)$test out$test <- x out}))
MI2$bonf <- p.adjust(MI2$p.value, method = “bonferroni”)
MI2[MI2$bonf <0.05,]
## Fit model with cross-loading freed
fit.cross <- cfa(c(mod.config, 'visual =~ x9'), data = HS, std.lv = TRUE, group = “school”)
summary(fit.cross, fit = TRUE)
anova(fit.config, fit.cross)
